# Edible red seaweed *Campylaephora hypnaeoides* J. Agardh alleviates obesity and related metabolic disorders in mice by suppressing oxidative stress and inflammatory response

**DOI:** 10.1186/s12986-021-00633-5

**Published:** 2022-01-08

**Authors:** Shigeru Murakami, Chihiro Hirazawa, Rina Yoshikawa, Toshiki Mizutani, Takuma Ohya, Ning Ma, Takahiko Ikemori, Takashi Ito, Chiaki Matsuzaki

**Affiliations:** 1 Department of Bioscience and Biotechnology, Fukui Prefectural University, Fukui, 9101195 Japan; 2 Division of Health Science, Graduate School of Health Science, Suzuka University, Mie, 5100293 Japan; 3 Ishikawa Prefecture Fisheries Division, Ishikawa, 9208580 Japan; 4 Research Institute for Bioscience and Biotechnology, Ishikawa Prefectural University, Ishikawa, 9218836 Japan

**Keywords:** Obesity, Diabetes, Hepatic steatosis, Seaweed, *Campylaephora hypnaeoides* J. Agardh, Polysaccharide, Oxidative stress, Inflammation

## Abstract

**Background:**

The obesity epidemic has become a serious public health problem in many countries worldwide. Seaweed has few calories and is rich in active nutritional components necessary for health promotion and disease prevention. The aim of this study was to investigate the effects of the *Campylaephora hypnaeoides* J. Agardh (*C. hypnaeoides*), an edible seaweed traditionally eaten in Japan, on high-fat (HF) diet-induced obesity and related metabolic diseases in mice.

**Methods:**

Male C57BL/6J mice were randomly divided into the following groups: normal diet group, HF diet group, HF diet supplemented with 2% *C. hypnaeoides,* and HF diet supplemented with 6% *C. hypnaeoides.* After 13 weeks of treatment, the weight of the white adipose tissue and liver, and the serum levels of glucose, insulin, adipokines, and lipids were measured. Hepatic levels of adipokines, oxidant markers, and antioxidant markers were also determined. Insulin resistance was assessed by a glucose tolerance test. Polysaccharides of *C. hypnaeoides* were purified and their molecular weight was determined by high-performance seize exclusion chromatography. The anti-inflammatory effects of purified polysaccharides were evaluated in RAW264.7 cells.

**Results:**

Treatment of HF diet-induced obese mice with *C. hypnaeoides* for 13 weeks suppressed the increase in body weight and white adipose tissue weight. It also ameliorated insulin resistance, hyperglycemia, hepatic steatosis, and hypercholesterolemia. The ingestion of an HF diet increased serum levels of malondialdehyde (MDA), tumor necrosis factor α (TNF-α), and monocyte chemoattractant protein-1 (MCP-1), while it decreased serum adiponectin levels. In the liver, an HF diet markedly increased the MDA, TNF-α, and interleukin-6 (IL-6) levels, while it decreased glutathione and superoxide dismutase. These metabolic changes induced by HF diet feeding were ameliorated by dietary *C. hypnaeoides.* Purified polysaccharides and ethanol extract from *C. hypnaeoides* inhibited the lipopolysaccharide-induced overproduction of nitric oxide and TNF-α in macrophage RAW264.7 cells.

**Conclusions:**

The present results indicated that *C. hypnaeoides* was able to alleviate HF diet-induced metabolic disorders, including obesity, hyperglycemia, hepatic steatosis, and hypercholesterolemia by attenuating inflammation and improving the antioxidant capacity in mice. Polysaccharides and polyphenols may be involved in these beneficial effects of *C. hypnaeoides*.

## Introduction

The prevalence of obesity has increased worldwide over the past decades. Obesity is a chronic and complex metabolic disease and is defined as a condition of abnormal or excessive fat accumulation in adipose tissue resulting in impaired health [1]. A lack of physical activity, sedentary lifestyle and energy-rich diet are the main causes of obesity. It is well established that obesity is the main risk factor for a number of non-communicable diseases, including type II diabetes, dyslipidemia, cardiovascular disease, hypertension, coronary heart disease, and certain types of cancers [2–4].

Seaweed has few calories and contains many different ingredients necessary for maintaining health, including minerals, vitamins, polysaccharides, polyunsaturated fatty acids, inorganic elements, and polyphenols [5]. Animal experiments have revealed the beneficial effects of seaweed and seaweed components on the prevention of diseases, including cardiovascular disease, metabolic disorders, neurodegenerative disease, and cancer [5, 6]. For example, various types of polysaccharides present in seaweed exhibit diverse biological and pharmacological activities, including antioxidant, immunomodulatory, anticoagulant, antitumor, antiviral and hypolipidemic activities [7, 8]. Furthermore, recent studies have shown that seaweed polysaccharides are resistant to gastrointestinal enzyme digestion and serve as fermentation substrates for intestinal microbial populations [9]. Seaweed is a rich source of phenolic compounds. The phenolic derivatives also exhibit a broad spectrum of biological properties, including antioxidant, anti-inflammatory, antimicrobial, anticancer, antidiabetic, and anti-obesity activities [10, 11]. These active components have been investigated for their potential use in food, cosmetic, and pharmaceutical applications [12]. Seaweed has long been used as an ingredient in Japanese food and is considered to be an ingredient that has supported the health and longevity of Japanese people. Epidemiological evidences among the Japanese indicates that the intake of seaweed is associated with a lower incidence of cardiovascular disease mortality [13, 14]. We previously demonstrated that some Japanese seaweeds prevented the development of metabolic disorders, such as obesity, diabetes, dyslipidemia, and hepatic steatosis in mice fed an HF diet [15].

*Campylaephora hypnaeoides* J. Agardh (*C. hypnaeoides*), an edible red seaweed also known as Egonori in Japan, grows along the coast of the Japan Sea. *C. hypnaeoides* is rich in polysaccharides, and after being boiled and hardened, it has long been eaten as a local dish in various parts of Japan. It is also used as a raw material for agar. We previously demonstrated that a 70% ethanol extract of *C. hypnaeoides* suppressed postprandial hyperglycemia by inhibiting the activity of carbohydrate-degrading enzymes [16]. It also ameliorated the high glucose-induced overproduction of reactive oxygen species and cell apoptosis through anti-oxidant and anti-inflammatory effects in cultured human umbilical vein endothelial cells [16]. Although a detailed analysis of constituents has not been performed, *C. hypnaeoides* is known to be rich in polysaccharides and polyphenols. From the results of these previous studies, *C. hypnaeoides* is expected to be useful for the prevention of metabolic disorders such as obesity and diabetes. The present study was carried out to determine the effects of *C. hypnaeoides* on the development of obesity and related metabolic disease in mice fed an HF diet.

## Methods

### The purification and molecular mass analysis of polysaccharides

The extraction of polysaccharides was performed according to a previously described method by a previously described procedure [17]. Briefly, 200 mg of powdered seaweed was incubated in 20 ml of 85% ethanol at 80 °C for 4 h to remove lipids. This extraction operation was repeated three times. Polysaccharides in the residue were extracted with 30 ml of cold water, dialyzed against distilled water, and then freeze-dried (32.0 mg). The total sugar content was determined by phenol–sulfuric acid method using galactose as a standard [18]. The sulfate content was determined by the BaCl_2_-gelatin method [19] using κ-carrageenan (TCI, Tokyo, Japan) as a positive control. The relative molecular weight of the polysaccharides was determined by high-performance size exclusion chromatography with Shodex OHpak SB-807G (Guard), SB-807 HQ, and SB-806 M HQ (8.0 mm ID × 300 mm length; Showa Denko KK, Tokyo, Japan) at 40 °C and estimated using dextran standards (150, 270, and 670 kDa from Sigma Aldrich Corp., 3,755 kDa from American Polymer Standards Corp., Mentor, OH, USA). Sample (injected volume: 20 μl) was eluted using 0.3 M NaNO_3_ at a flow rate of 1 ml/min and was detected using a refractive index (RI) detector RID10 (Shimadzu Corp., Kyoto, Japan).

### Preparation of polyphenol extract and measurement of polyphenols

Powdered *C. hypnaeoides* were extracted with 80% ethanol at room temperature for 48 h. The ethanol extract was filtered and concentrated using a rotary evaporator. Total polyphenols were determined by a previously reported method with slight modifications [20]. Phenolic extract (10 μl) was placed in triplicate in a 96-well plate, and mixed with 25 μl of 1 N Folin-Ciocalteu reagent and 20% sodium bicarbonate, followed by 150 μl distilled water. The mixture was incubated for 30 min at room temperature before recording the absorbance at 630 nm. Total polyphenol was calculated according to the gallic acid standard curve. The total polyphenol content in ethanol extract was 9.65 mg gallic acid equivalent/g.

### Animals and dietary treatment

Male C57BL/6 J mice (6-week-old) were purchased from CLEA Japan Inc. (Tokyo, Japan) and housed at a constant temperature of 22 ± 1 °C with a 12 h light/dark cycle. After 1 week of acclimation, the animals were randomly divided into 4 groups of 12–13 animals, as follows: (1) normal diet (normal) group, high-fat (HF) diet group, HF diet supplemented with 2% *C. hypnaeoides* (HF + ChL) group, and HF supplemented with 6% *C. hypnaeoides* (HF + ChH) group. The doses of *C. hypnaeoides* were decided based on the previous studies [15]. The *C. hypnaeoides* sample was harvested on the coast of Niigata Prefecture and was washed with water. It was dried, reduced to a fine powder using a food mixer, and mixed into HF chow. The composition of the experimental diets was adjusted by considering the nutritional components of *C. hypnaeoides* as described previously [15]. The normal diet provided 354 kcal/100 g of energy (14.4% calories from protein, 11.1% calories from fat and 74.4% calories from carbohydrate), while the HF diet provided 493 kcal/100 g of energy (17.9% calories from protein, 60.7% calories from fat and 21.4% calories from carbohydrate). All experimental diets were based on the AIN-76 diet (Oriental Yeast Co. Ltd., Tokyo, Japan). Animals were allowed ad libitum access to food and drinking water. The food, including the HF diet was exchanged with new food every day. The body weight and food intake were monitored twice a week. After 13 weeks of feeding, the mice were deprived of food overnight, and blood samples were withdrawn under mixed anesthetic agent (0.3 mg/kg of medetomidine, 4.0 mg/kg of midazolam, and 5.0 mg/kg of butorphanol; Fujifilm Wako Pure Chemical Co., Osaka, Japan). Liver tissue and epididymal, peritoneal, and mesenteric white adipose tissues were removed, weighed, and stored at −80 °C. Some mice were used for histological examinations. All experimental protocols were approved by the Institutional Animal Care and Use Committee of Fukui Prefectural University (Approval No. 19-14).

### Serum biochemical analyses

Serum was obtained by centrifugation at 1500×*g* for 15 min at 4 °C. The serum levels of total cholesterol, high-density lipoprotein (HDL)-cholesterol, triglyceride, alanine aminotransferase (ALT) and aspartate aminotransferase (AST) were analyzed using a Hitachi 7060 Automatic Analyzer (Hitachi, Tokyo, Japan) with commercial kits (Fujifilm Wako Pure Chemical Co., Osaka, Japan). The serum insulin (Morinaga Institute of Biological Science, Yokohama, Japan), adiponectin (Otsuka Pharmaceutical Co. Ltd. Tokyo, Japan), tumor necrosis factor-α (TNF-α; Fujifilm Wako Pure Chemical Co., Osaka, Japan), and monocyte chemoattractant protein-1 (MCP-1; Proteintech, Rosemont, IL, USA) levels were also determined using a commercial ELISA kit. Serum malondialdehyde (MDA) levels were determined using a commercial kit (Japan Institute for the Control of Aging Co. Ltd., Shizuoka, Japan). The homeostasis model assessment insulin resistance (HOMA-IR), an insulin resistance index, was calculated using the following equation: HOMA-IR = fasting glucose (mg/dL) × fasting insulin (ng/mL)/22.5.

### Hepatic biochemical analysis

Lipids were extracted from the liver according to a previously described method [21]. In brief, frozen liver tissues (50 mg) were homogenized in 5 volumes of isopropanol. The homogenate was kept at room temperature for 2 days and then centrifuged at 1000×*g* for 10 min. Aliquots of supernatant were analyzed for triglyceride content using a commercial kit (Fujifilm Wako Pure Chemical Co., Osaka, Japan). Liver samples for the biochemical analysis were homogenized in 5 volumes of cold 20 mM Tris–HCl buffer (pH 7.4), and centrifuged at 12,000×*g* for 15 min at 4 °C. The supernatant was used for the biochemical analysis. Lipid peroxidation was determined by estimating malondialdehyde (MDA) using a commercial kit (Japan Institute for the Control of Aging Co. Ltd., Shizuoka, Japan). The reduced glutathione (GSH) levels were determined using a commercial kit (Japan Institute for the Control of Aging Co. Ltd., Shizuoka, Japan). The inflammatory cytokine levels in the liver were quantified using enzyme-linked immunosorbent assay (ELISA) kits specific for mouse TNF-α and interleukin-6 (IL-6). (Fujifilm Wako Pure Chemical Co., Osaka, Japan). The protein concentration was determined using a Quick Start Bradford protein assay (Bio-Rad Laboratories, Inc., Hercules, CA, USA).

### Histological analyses

The mice were anesthetized with an intraperitoneal injection of mixed anesthetic agent (0.3 mg/kg of medetomidine, 4.0 mg/kg of midazolam, and 5.0 mg/kg of butorphanol; Fujifilm Wako Pure Chemical Co., Osaka, Japan), and transcardially perfused with a fixative containing 4% paraformaldehyde and 1.5% glutaraldehyde in phosphate-buffered saline (PBS). After perfusion, the liver and white adipose tissue were removed and allowed to stand in the same fixative for one day. The tissues were rinsed several times with PBS and embedded in paraffin. Tissues were cut into 5-μm-thick sections, mounted on slides, and stained with hematoxylin eosin (HE).

### Glucose tolerance test

A glucose loading test was performed one week before the end of experiment to access glucose intolerance [22]. The mice were fasted overnight and intraperitoneally injected with glucose (2 g/kg body weight). Blood samples were collected from the tail veins of the mice, and glucose levels were measured at 0, 30, 60, 90, and 120 min after injection using a blood glucometer Nipro Stat Strip (Nipro, Osaka, Japan).

### Fecal analyses

During fecal collection, mice were separated, and fecal samples were collected for a 24-h period from each mouse and weighed. These samples were ground into a powder in a mortar, and 50 mg of feces was extracted with 300 μl of distilled water. After centrifugation (16,000×*g*, 30 min, 4 °C), ethanol was added to the supernatant (final concentration of 85%), and polysaccharides were obtained as the precipitate. The resulting residue was washed with 85% ethanol and dried. The residue was then resuspended in distilled water and centrifuged (16,000×*g*, 10 min, 4 °C), and the polysaccharide content in the supernatant was measured using the phenol–sulfuric acid method, which has been described elsewhere, with galactose as the standard [18]. For the measurement of triglycerides, lipids were extracted by adding isopropanol (10 times the weight) to the fecal powder. The sample was then dried and dissolved in isopropanol. The triglyceride concentration was measured using a commercial kit (Fujifilm Wako Pure Chemical Co., Osaka, Japan).

### Nitric oxide and cytokine assays

RAW264.7 cells were prepared to a concentration of 2 × 10^5^ cells/ml using MEM medium (M5650, Sigma Aldrich) supplemented with 10% fetal bovine serum and antibiotics (penicillin 100 U/ml and streptomycin 100 μl/ml) The cell suspension was added to a 96-well plate and cultured in a 5% CO_2_ incubator for 24 h. After washing each well with phosphate-buffered saline, lipopolysaccharide (LPS; derived from *Escherichia coli* O111, Fujifilm Wako Pure Chemical Co., Osaka, Japan) with a final concentration of 100 ng/mL and polysaccharide or ethanol extract solution were added to the medium. The cells were cultured in a 5% CO_2_ incubator for an additional 24 h. The culture supernatant (100 μl) was collected, 100 μl of Griess reagent was added, and the mixture was left for 20 min in the dark; the absorbance was then measured by microplate reader at 543 nm. The nitric oxide concentration in the medium was calculated from the standard curve prepared from sodium nitrite. Cell viability was evaluated via a 3- (4,5-dimethylthial-2-yl) -2,5-diphenyltetrazalium bromide (MTT) colorimetric assay.

### Statistical analysis

Results are expressed as the mean ± SEM. Data were analyzed by a one-way analysis of variance (ANOVA) followed by Turkey’s multiple range tests. *P* values of *p* < 0.05 were considered to indicate statistical significance.

## Results

### Isolation of polysaccharide

The yield of the obtained *C. hypnaeoides* polysaccharides was 16.0%. It showed a symmetric peak on high-performance size exclusion chromatography and the molecular mass was estimated to be 1.4 × 10^5^ Da. The polysaccharides contained 95.8% sugar and 3.3% sulfate. The sulfate content of *C. hypnaeoides* was much lower than that of κ-carrageenan (17.8%), which was used as a positive control.

### Food intake and body weight

**Fig. 1:**
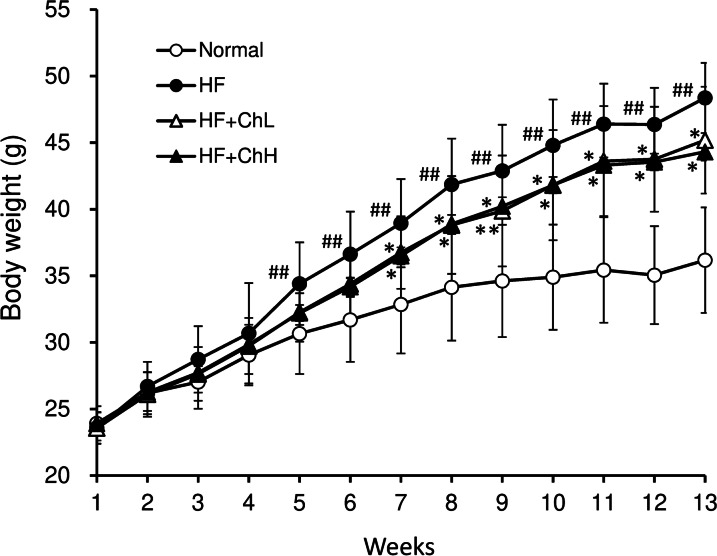
The effects of *C. hypnaeoides* on body weight in C57BL/6J mice fed a high-fat diet. Normal, normal diet; HF, high-fat diet; HF + ChL, high-fat diet mixed with 2% *C. hypnaeoides*; HF + ChH, high-fat diet mixed with 6% *C. hypnaeoides.* Each value represents the mean ± SD (n = 12–13). Statistical significance: *p < 0.05 vs. HF group, ^##^*p* < 0.01 versus Normal group

The addition of 2% or 6% *C. hypnaeoides* to an HF diet did not affect the amount of food consumption. The average daily food intake of each group throughout the experimental period was as follows: 3.1 g (Normal), 2.5 g (HF), 2.5 g (HF + ChL), and 2.6 g (HF + ChH). The energy intake of mice in the normal group was not significantly different from that of mice in the HF group (12.1 vs. 12.8 kcal/mouse/day). HF diet feeding to mice for 13 weeks induced marked body weight gain, which was double that of normal mice (Table 1). The body weights began to differ significantly between the HF group and the normal group after 5 weeks of treatment, and between the HF group and HF + ChH group after 7 weeks of treatment (Fig. 1). At the end of experiment, the body weight of both the HF + ChL and HF + ChH groups was significantly lower in comparison to the mice in the HF group.

**Table 1:** The effects of *C. hypnaeoides* on the body weight gain and serum metabolic parameters in C57BL/6 J mice fed a high-fat diet

Variables	Normal	HF	HF + ChL	HF + ChH
Body weight gain	12.3 ± 3.5	24.8 ± 1.9^##^	21.6 ± 3.7*	20.4 ± 2.1*
Glucose (mg/dL)	97 ± 13	155 ± 12^##^	104 ± 13**	98 ± 15**
Insulin (ng/mL)	0.51 ± 0.14	2.50 ± 0.90^##^	0.80 ± 0.32**	0.63 ± 0.05**
HOMA-IR	3.45 ± 1.3	26.9 ± 8.1^##^	5.76 ± 2.3**	4.32 ± 0.51**
TC (mg/dL)	98 ± 19	187 ± 8^##^	173 ± 27	169 ± 7*
HDL-C (mg/dL)	58 ± 16	70 ± 7	73 ± 3	71 ± 4
TG (mg/dL)	47 ± 16	35 ± 9	39 ± 11	34 ± 6
ALT (IU/L)	24 ± 9	96 ± 27^##^	51 ± 35*	52 ± 20**
AST (IU/L)	89 ± 13	130 ± 29^##^	111 ± 24	102 ± 19*

*HOMA-IR* homeostatic model assessment-insulin resistance, *TC* total cholesterol, *HDL-C* high-density lipoprotein cholesterol, *TG* triglyceride, *ALT* alanine aminotransferase, *AST* aspartate aminotransferase

Data were expressed as mean ± SD (n = 6–12). Statistical significance: ^##^*p* < 0.01 vs. Normal group, *p < 0.05, **p < 0.01 versus HF group

### Body fat

**Fig. 2:**
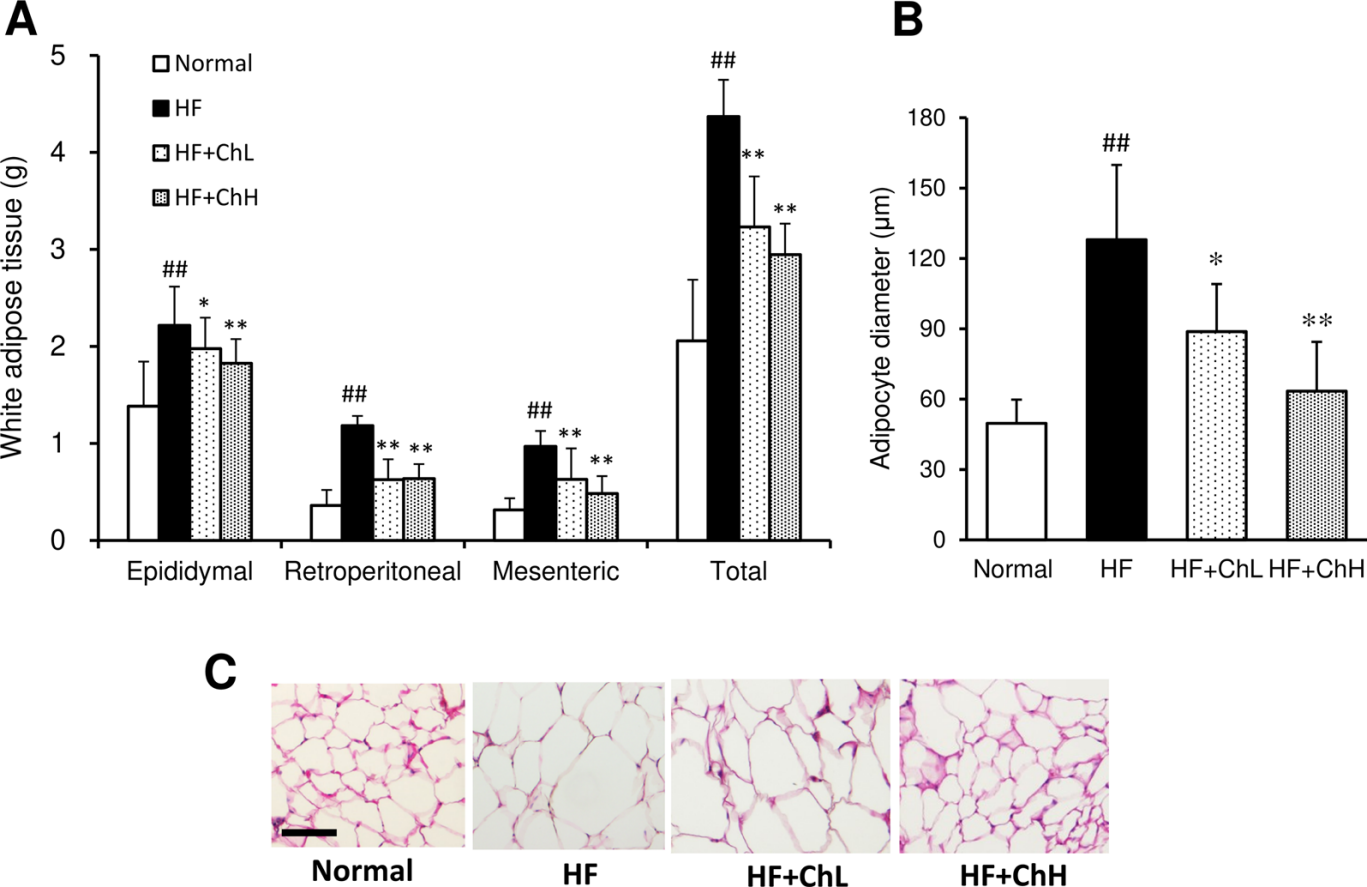
The effects of *C. hypnaeoides* on the mass and morphology of white adipose tissue in C57BL/6J mice fed a high-fat diet. After the mice were sacrificed, the mass of the epididymal, retroperitoneal, and mesenteric white adipose tissue was determined (**A**). Adipose tissue was fixed and the section of epididymal adipose tissue was stained with hematoxylin and eosin (**C**). The average diameter of adipocytes was determined (**B**). Normal, normal diet; HF, high-fat diet; HF + ChL, high-fat diet mixed with 2% *C. hypnaeoides*; HF + ChH, high-fat diet mixed with 6% *C. hypnaeoides*. Each value represents the mean ± SD (n = 8). Statistical significance: *p < 0.05, **p < 0.01 versus HF group, ^##^*p* < 0.01 versus Normal group. Scale bar = 100 μm

The weight of each adipose tissue (epididymal, retroperitoneal, and mesenteric) was significantly higher in the HF group than that in the normal group (Fig. 2A). The weight of total adipose tissue in the HF group was also significantly higher than that in the normal group. Thirteen-week-treatment of HF mice with *C. hypnaeoides* significantly reduced the total tissue weight and the weight of each adipose tissue (Fig. 2A). A histological analysis showed that adipocytes in HF mice were hypertrophic in comparison to those in normal mice (Fig. 2C). The diameter of adipocytes was significantly increased by administration of an HF diet. (Fig. 2B).In contrast, the adipocyte size was decreased by *C. hypnaeoides* supplementation (Fig. 2B, C).

### Serum levels of glucose and insulin, and insulin resistance

**Fig. 3:**
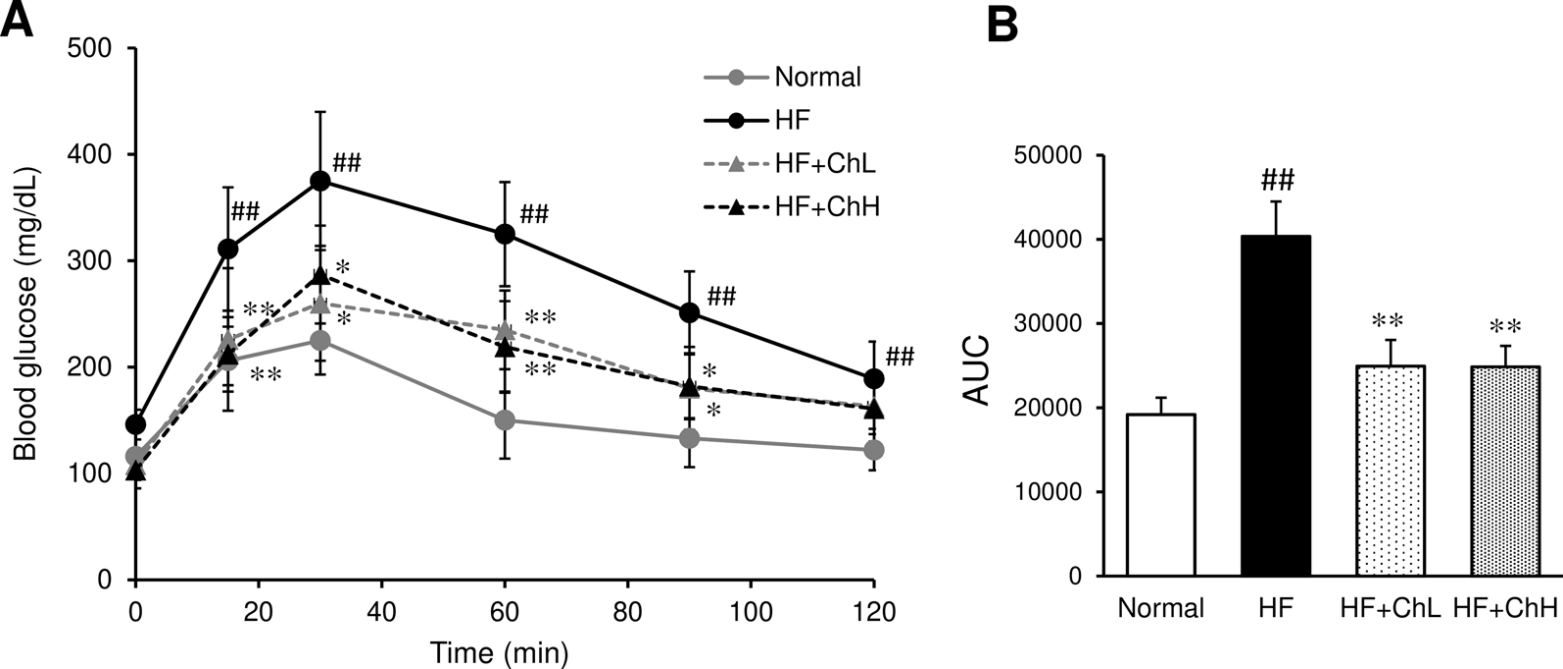
The effects of *C. hypnaeoides* on the insulin resistance in C57BL/6J mice fed a high-fat diet. Insulin resistance was evaluated by a glucose tolerance test in the 12th week after high-fat diet ingestion (**A**), and the area under the curve (AUC) was calculated (**B**). Normal, normal diet; HF, high-fat diet; HF + ChL: high-fat diet mixed with 2% *C. hypnaeoides*; HF + ChH: high-fat diet mixed with 6% *C. hypnaeoides*. Each value represents the mean ± SD (n = 6–8). Statistical significance: *p < 0.05, **p < 0.01 versus HF group, ^##^*p* < 0.01 versus Normal group

Although the ingestion of an HF diet increased the serum levels of glucose and insulin, *C. hypnaeoides* supplementation significantly suppressed the elevation of glucose and insulin levels (Table 1). The effects of *C. hypnaeoides* supplementation on insulin resistance were determined using a glucose tolerance test. The blood glucose levels in *C. hypnaeoides*-supplemented mice were lower at 15, 30, 60, and 90 min after the intraperitoneal administration of glucose, in comparison to mice in the HF group (Fig. 3A). The area under the curve (AUC) for glucose was also significantly lower in *C. hypnaeoides*-supplemented mice in comparison to mice in the HF group (Fig. 3B). The HOMA-IR value, which is an index of insulin resistance, was markedly increased by ingestion of an HF diet, but was ameliorated by *C. hypnaeoides* supplementation (Table 1).

### Hepatic lipid accumulation and serum liver function parameters

**Fig. 4:**
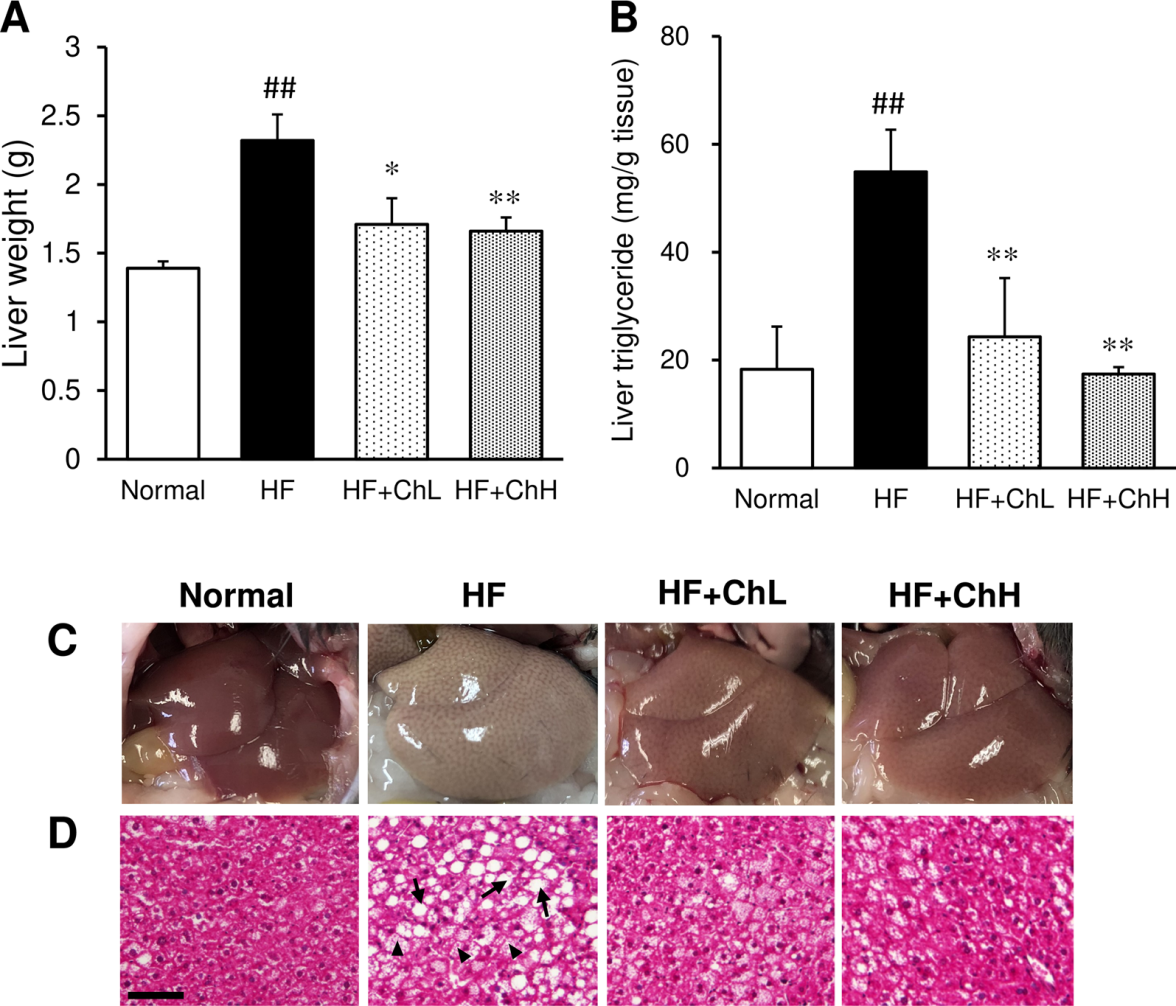
The effects of *C. hypnaeoides* on the hepatic lipid accumulation in C57BL/6J mice fed a high-fat diet. **A** Liver weight, **B** Triglyceride content, **C** Representative gross morphology, **D** Histological sections of the liver. Normal, normal diet; HF, high-fat diet; HF + ChL, high-fat diet mixed with 2% *C. hypnaeoides*; HF + ChH, high-fat diet mixed with 6% *C. hypnaeoides*. Each value represents the mean ± SD (n = 6–8). Arrows indicate lipid droplets, and arrow heads indicate hepatocyte swelling. Statistical significance: *p < 0.05, **p < 0.01 versus HF group, ^##^*p* < 0.01 versus Normal group. Scale bar = 100 μm

The feeding an HF diet to mice for 13 weeks markedly increased the liver triglyceride content, which was accompanied by an increase in liver weight (Fig. 4A, B). In HF diet-fed mice, supplementation with *C. hypnaeoides* suppressed the increase in liver weight and hepatic triglyceride content (Fig. 4A, B). In addition, elevated serum liver function parameters, including alanine aminotransferase (ALT) and aspartate aminotransferase (AST) were decreased by supplementation with *C. hypnaeoides* (Table 1)*.* The gross morphology of the liver showed that the liver of the mice in the HF group was larger and exhibited a paler color in comparison to the mice in the normal group (Fig. 4C). A histological examination revealed hepatic steatosis in the liver of mice in the HF group, as evidenced by vacuoles, lipid droplets, and hepatocyte swelling (Fig. 4D). The development of HF-induced hepatic steatosis was ameliorated by *C. hypnaeoides* supplementation.

### Hepatic parameters of oxidative stress and inflammation

The feeding an HF diet to mice increased the hepatic levels of malondialdehyde (MDA), an oxidative stress marker, while it decreased the hepatic levels of anti-oxidant defense markers, glutathione (GSH) and superoxide dismutase (SOD) (Table 2). An HF diet also increased hepatic levels of pro-inflammatory cytokines, TNF-α and IL-6 (Table 2). These changes in hepatic parameters recovered with *C. hypnaeoides* supplementation in a dose-dependent manner.

**Table 2:** The effects of *C. hypnaeoides* on the oxidant, anti-oxidant, and inflammatory markers of the serum and liver in C57BL/6 J mice fed a high-fat diet

Variables	Normal	HF	HF + ChL	HF + ChH
**Serum**				
MDA (nmol/mL)	1.51 ± 0.17	2.64 ± 0.47^##^	2.11 ± 0.31*	1.85 ± 0.37**
adiponectin (μg/mL)	22.8 ± 0.97	16.2 ± 1.4^##^	16.7 ± 3.0	20.9 ± 1.8*
TNF-α (pg/mL)	4.21 ± 1.4	10.66 ± 3.3^##^	8.93 ± 1.7	5.93 ± 2.1*
MCP-1 (pg/mL)	32.7 ± 5.8	45.0 ± 9.7^#^	35.2 ± 7.3*	26.4 ± 5.9*
**Liver**				
MDA (nmol/mg protein)	6.53 ± 1.3	16.1 ± 4.3^##^	11.3 ± 2.8 *	10.3 ± 2.0**
GSH (nmol/mg protein)	81.8 ± 15	59.3 ± 18^#^	65.3 ± 18	77.2 ± 13*
SOD (U/mg protein)	8.34 ± 1.5	5.54 ± 1.5^##^	6.21 ± 2.3	9.15 ± 3.3*
TNF-α (pg/mg protein)	5.14 ± 1.2	12.35 ± 3.2^##^	7.67 ± 1.6**	6.51 ± 1.4**
IL-6 (pg/mg protein)	1.47 ± 0.21	2.18 ± 0.45^##^	1.99 ± 0.44	1.63 ± 0.28*

*MDA* malondialdehyde, *TNF-α* tumor necrosis factor α, *MCP-1* monocyte chemoattractant protein-1, *GSH* glutathione, *SOD* superoxide dismutase

Data were expressed as mean ± SD (n = 6–8). Statistical significance: ^#^*p* < 0.05, ^##^*p* < 0.01 versus Normal group, *p < 0.05, **p < 0.01 versus HF group

### Serum parameters of oxidative stress and inflammation

Consistent with the effects of *C. hypnaeoides* on hepatic parameters of oxidative stress and inflammation, the HF-induced elevation of serum MDA, TNF-α, and monocyte chemoattractant protein-1 (MCP-1) levels was ameliorated by *C. hypnaeoides* supplementation in a dose-dependent manner (Table 2). In addition, the HF-induced decrease in serum adiponectin levels was suppressed by *C. hypnaeoide*s (Table 2).

### Serum lipid levels

The ingestion of an HF diet markedly increased levels of serum total cholesterol, which was significantly suppressed by a high dose of *C. hypnaeoides* (Table 1). Similar to previous study, the feeding of an HF diet had no significant effects on the serum levels of HDL cholesterol or triglyceride (Table 1) [15]. *C. hypnaeoides* supplementation also had no effect on these lipids.

### Fecal levels of lipids and polysaccharides

**Fig. 5:**
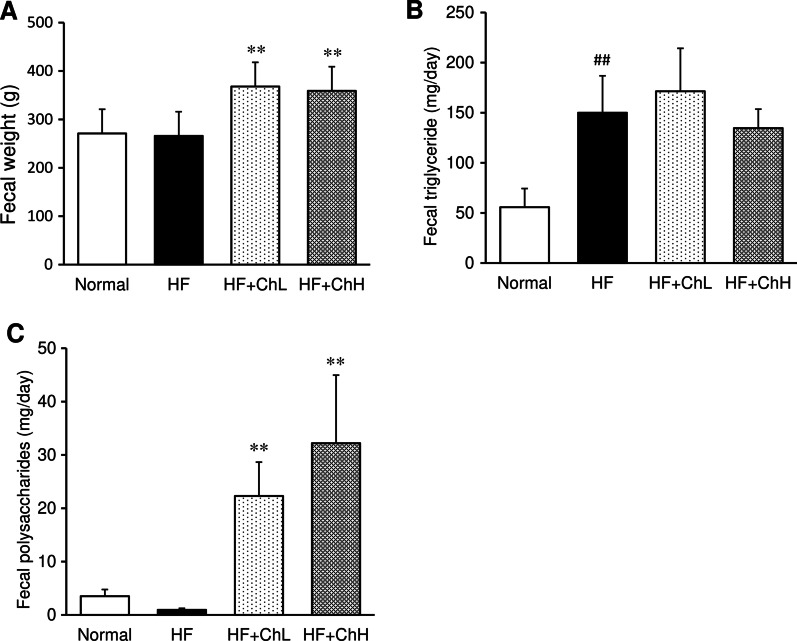
The effects of *C. hypnaeoides* on the fecal weight and fecal components in C57BL/6J mice fed a high-fat diet. Feces samples were collected from each mouse after high-fat diet ingestion for 10 weeks, and the fecal weight was measured (**A**). The triglyceride (**B**) and polysaccharides (**C**) content were determined after extraction with isopropanol and distilled water, respectively. Normal, normal diet; HF, high-fat diet; HF + ChL, high-fat diet mixed with 2% *C. hypnaeoides*; HF + ChH, high-fat diet mixed with 6% *C. hypnaeoides*. Each value represents the mean ± SD (n = 7). Statistical significance: **p < 0.01 versus HF group, ^##^*p* < 0.01 versus Normal group

The fecal weight and fecal polysaccharide content were significantly increased by *C. hypnaeoides* supplementation (Fig. 5A, C)*.* Although the HF increased the fecal triglyceride content, *C. hypnaeoides* supplementation had no effects (Fig. 5B).

#### Nitric oxide and TNF-α production in RAW264.7 cells

**Fig. 6:**
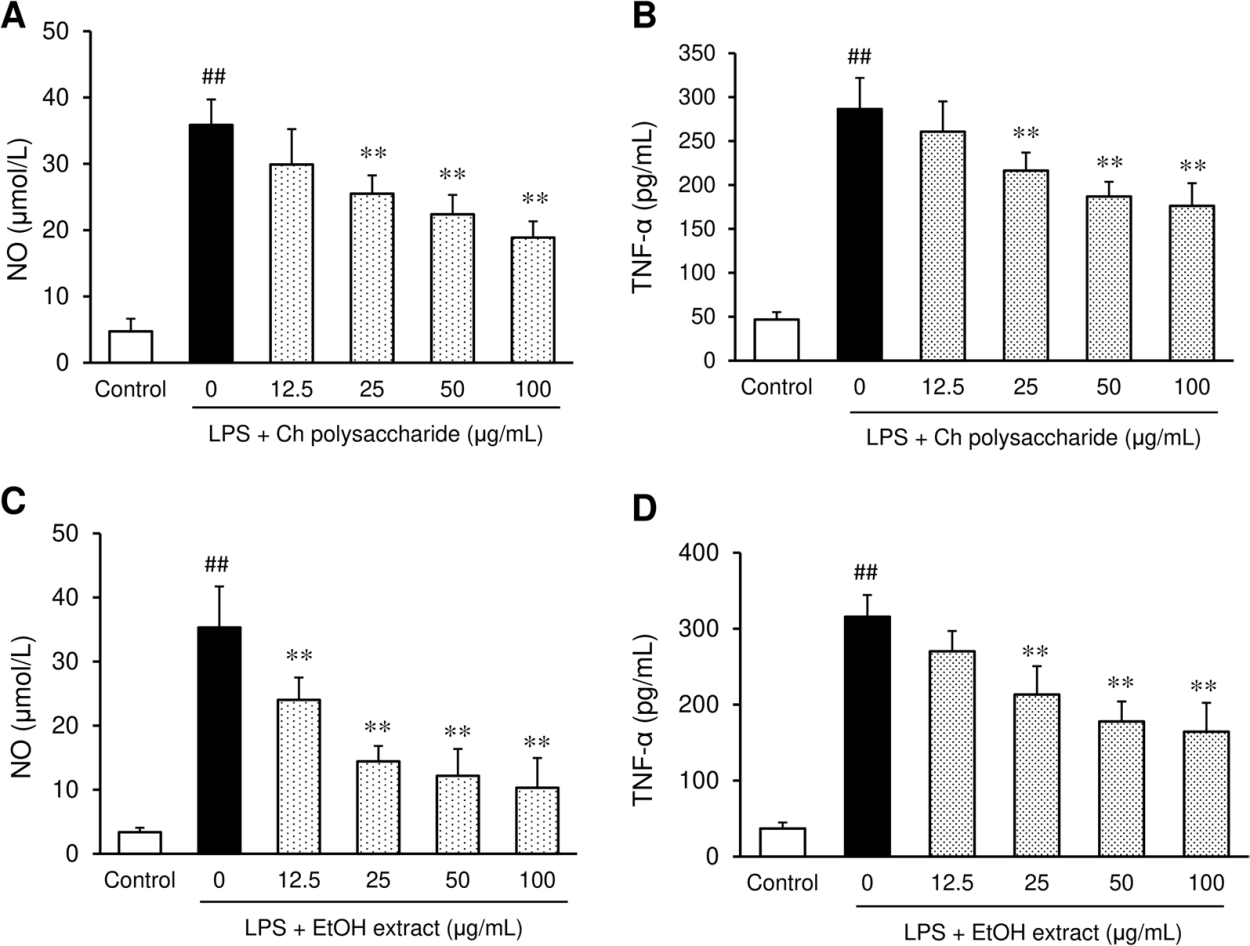
The effects of *C. hypnaeoides* polysaccharides and ethanol extract on the production of nitric oxide (**A**, **C**) and TNF-α (**B**, **D**) in RAW 264.7 cells. Each values represents the mean ± SD (n = 6). Statistical significance: **p < 0.01 versus LPS + Ch polysaccharides (0 μg/ml) or LPS + EtOH extract (0 μg/ml), ^##^*p* < 0.01 versus Control

The anti-inflammatory effects of polysaccharides isolated from *C. hypnaeoides* were assessed in macrophage RAW264.7 cells. The addition of polysaccharides to the culture medium suppressed the lipopolysaccharide (LPS)-induced production of nitric oxide (NO) and TNF-α in a dose-dependent manner (Fig. 6A, B). Ethanol extract also suppressed the LPS-induced production of NO and TNF-α (Fig. 6C, D).

## Discussion

The present study showed that an edible red seaweed, *C. hypnaeoides* exhibited an anti-obesity effect in mice fed a high-fat (HF) diet, as was evident from the reduced body weight gain and reduced body fat accumulation. In addition, *C. hypnaeoides* ameliorated obesity-associated metabolic disorders, including insulin resistance, hyperglycemia, hepatic steatosis, and hypercholesterolemia. The beneficial effects of *C. hypnaeoides* were associated with a reduction of oxidative stress and inflammation. It is well documented that oxidative stress and inflammation are closely involved in various kinds of diseases, including obesity and obesity-related noncommunicable diseases [23]. The excessive intake of major nutrients such as an HF diet activates metabolic signaling pathways, including c-Jun N-terminal kinase (JNK) and nuclear factor-κB (NF-κB), and leads to the induction of inflammatory cytokines, resulting in a low-grade inflammatory response. A wide variety of naturally occurring compounds and extracts have been shown to ameliorate obesity, diabetes, and hepatic steatosis through anti-oxidative and anti-inflammatory effects [24, 25], although there is fewer information from studies in humans in comparison to studies using animal models [26].

Adipose tissue is not merely a simple reservoir of energy stored as triglycerides, it also serves as an active secretory organ releasing many physiologically active substances, referred to as adipokines, into the circulation [27]. During the development of obesity, adipocytes become hypertrophic and the balance between adipokines is altered. Hypertrophic adipocytes produce more pro-inflammatory adipokines, such as TNF-α and IL-6, and fewer anti-inflammatory adipokines, such as adiponectin in comparison to normal adipocytes [28]. In addition, inflammatory cells such as leukocytes, macrophages, and T-cells recruit the surrounding adipocytes with lipid accumulation in adipocytes. Monocyte chemoattractant protein–1 (MCP-1) is produced by macrophages and endothelial cells and is a potent chemotactic factor for monocytes [29]. Circulating levels of TNF-α and IL-6 have been shown to be correlated with increasing adipose mass [30]. The plasma level and adipose tissue expression of MCP-1 have also been found to be increased in obese humans [31] and animals [32] in comparison to lean controls. Elevated circulating pro-inflammatory adipokines induce peripheral insulin resistance [33]. Adiponectin is a major anti-inflammatory adipokine, which is produced exclusively by adipocytes and which exhibits a wide range of biological activities, including anti-inflammatory, insulin-sensitizing, anti-atherogenic, and cardioprotective properties [34]. The circulating levels of adiponectin in obese patients and patients with diabetes are lower in comparison to healthy volunteers [35]. A negative association between serum adiponectin levels and the body mass index has been reported in diabetic patients. Therefore, a decrease in serum adiponectin levels predicts the development of metabolic disorders, such as insulin resistance and type II diabetes [36]. Consistent with these previous observations, the serum levels of TNF-α, IL-6, and MCP-1 were elevated in obese mice fed an HF diet, while the level of adiponectin was decreased. The treatment of obese mice with *C. hypnaeoides* recovered serum adipokines to the normal levels in a dose-dependent manner. In addition, *C. hypnaeoides* suppressed the HF-induced elevation of both TNF-α and IL-6 levels in the liver. An elevated inflammation response is known to be related to the onset and development of metabolic disorders other than obesity, including insulin resistance, diabetes, hepatic steatosis, and atherosclerosis. Thus, the anti-inflammatory action is one possible mechanism by which *C. hypnaeoides* ameliorated metabolic diseases in HF-induced obese mice.

Reactive oxygen species (ROS) play an important role in physiological cellular processes, and their imbalance and excessive production leads to oxidative stress, followed by damage to biomolecules such as lipids, proteins, and DNA [37]. Biologically, the body employs a range of antioxidant defense system to counter excessive oxidative stress, which includes superoxide dismutase (SOD) and catalase. Glutathione is a major non-protein antioxidant that protects cells against oxidative injury by scavenging ROS. Reduced glutathione levels are associated with the pathogenesis of many diseases, including liver disease and diabetes [38]. It has been shown that obese individuals exhibit higher levels of oxidative stress in adipose tissue, including elevated ROS production and decreased antioxidant activity [39]. In the present study, hepatic and serum levels of MDA, a biomarker of oxidative stress, were markedly elevated in obese mice, while hepatic glutathione and SOD levels were reduced in the liver of obese mice in comparison to normal mice, suggesting increased oxidative stress and a decreased antioxidant defense system. The treatment of obese mice with *C. hypnaeoides* reduced the MDA levels and increased the glutathione and SOD in the liver in a dose-dependent manner. These findings indicate that oxidative stress was increased in obese mice and that *C. hypnaeoides* ameliorated obesity and obesity-related metabolic disorders through an antioxidative mechanism. We have previously demonstrated the anti-inflammatory and anti-oxidant actions of 70% ethanol extract of *C. hypnaeoides* in cultured cells and in vitro [16]. Ethanol extract of *C. hypnaeoides* containing rich polyphenols exhibited a potent anti-oxidative effect and inhibited cell apoptosis via the suppression of ROS production in human umbilical vein endothelial cells. It also inhibited lipopolysaccharide (LPS)-induced nitric oxide (NO) production in RAW 264.7 cells, indicating anti-oxidant and anti-inflammatory activity. The present study showed that polysaccharides isolated from *C. hypnaeoides* suppressed LPS-induced production of NO and TNF-α in RAW264.7 cells. These results suggest that polysaccharide and polyphenol may be involved in the anti-oxidant and anti-inflammatory mechanisms by which *C. hypnaeoides* improved metabolic dysfunction.

We have previously shown that extracts of some seaweeds inhibited the activity of pancreatic lipase and suppressed the absorption of triglyceride. As a result, the fecal triglyceride content was increased [15]. As pancreatic lipase is a key enzyme that catalyzes the hydrolysis of triglyceride to monoacylglycerols and fatty acids, the inhibition of this enzyme activity results in reduced lipid absorption and subsequent amelioration of body fat accumulation. The treatment of mice in the HF group with *C. hypnaeoides* had no significant effects on fecal triglyceride content, suggesting that the inhibition of intestinal lipid absorption is not involved in the anti-obesity effect of *C. hypnaeoides.*

## Conclusion

In summary, this study showed that the Japanese traditional edible seaweed *C. hypnaeoides,* ameliorated obesity, hyperglycemia, hepatic steatosis, and hypercholesterolemia in obese mice fed an HF diet. The beneficial effects of *C. hypnaeoides* on HF-induced metabolic disorders were associated with reduced oxidative stress and inflammation. Polysaccharides and polyphenols may play an important role in preventing these disorders. The possible effects of *C. hypnaeoides* in obese and diabetic individuals need to be further investigated.

## Data Availability

The datasets used and/or analyzed during the current study are available from the corresponding author on reasonable request.

## Abbreviations

ALT: Alanine aminotransferase
AST: Aspartate aminotransferase
AUC: Area under the curve
*C. hypnaeoides*: *Campylaephora hypnaeoides* J. Agardh
HF: High-fat diet
GSH: Glutathione
HDL: High-density lipoprotein
IL-6: Interleukin-6
MDA: Malondialdehyde
MCP-1: Monocyte chemoattractant protein-1
NO: Nitric oxide
ROS: Reactive oxygen species
SOD: Superoxide dismutase
TNF-α: Tumor necrosis factor-α
